# Poster Session II - A225 SYSTEMIC INFLAMMATORY PATHWAYS LINK LOW BRAIN CORTICAL OXYGENATION TO SYMPTOM SEVERITY IN CROHN’S DISEASE

**DOI:** 10.1093/jcag/gwaf042.225

**Published:** 2026-02-13

**Authors:** A Soroush, A Hansen, C Ma, C Diribe, A Fuhrmann, D Marshall, C Lu, C Seow, R Ingram, K Novak, G G Kaplan, R Panaccione, J F Dunn, M G Swain

**Affiliations:** University of Calgary Cumming School of Medicine, Calgary, AB, Canada; University of Calgary Cumming School of Medicine, Calgary, AB, Canada; University of Calgary Cumming School of Medicine, Calgary, AB, Canada; University of Calgary Cumming School of Medicine, Calgary, AB, Canada; University of Calgary Cumming School of Medicine, Calgary, AB, Canada; University of Calgary Cumming School of Medicine, Calgary, AB, Canada; University of Calgary Cumming School of Medicine, Calgary, AB, Canada; University of Calgary Cumming School of Medicine, Calgary, AB, Canada; University of Calgary Cumming School of Medicine, Calgary, AB, Canada; University of Calgary Cumming School of Medicine, Calgary, AB, Canada; University of Calgary Cumming School of Medicine, Calgary, AB, Canada; University of Calgary Cumming School of Medicine, Calgary, AB, Canada; University of Calgary Cumming School of Medicine, Calgary, AB, Canada; University of Calgary Cumming School of Medicine, Calgary, AB, Canada

## Abstract

**Background:**

Patients with Crohn’s disease (CD) experience altered interoception and poor sleep quality. The prefrontal cortex (PFC), which regulates both processes, shows low oxygenation, quantified by tissue oxygen saturation (S_t_O_2_) using frequency-domain near-infrared spectroscopy (FD-NIRS), and has been linked to inflammation-related symptoms in Multiple Sclerosis and Long-COVID. However, this relationship has not been explored in CD, and its mechanisms remain unclear.

**Aims:**

To assess the impact of CD on PFC S_t_O_2_ and examine its associations with patient-reported outcome (PROs) and circulating biomarkers.

**Methods:**

Healthy controls (HC; *n*=19, age 41±15) and CD patients categorized by the Harvey–Bradshaw Index into remitted (rCD; *n*=16, age 51±14) and active disease (aCD; *n*=10, age 47±15) were recruited. Resting-state PFC S_t_O_2_ was measured using FD-NIRS. CD patients completed the Multidimensional Assessment of Interoceptive Awareness (MAIA) and Pittsburgh Sleep Quality Index (PSQI) questionnaires. Plasma cytokine/chemokine levels were quantified using multiplex assays. Between-group S_t_O_2_ differences were tested using ANCOVA (age-controlled). Spearman and Pearson correlations assessed S_t_O_2_ associations with PROs and cytokines, respectively, and mediation analyses evaluated pathways linking S_t_O_2_ and PROs with cytokines as mediators.

**Results:**

PFC S_t_O_2_ was significantly lower in the rCD patients compared to HCs (*p*=0.017; Table 1). Across all CD patients, S_t_O_2_ showed a moderate negative association with the MAIA *attention regulation* subscale (ignoring, distraction) (*ρ*=-0.63, *p*=0.004) and PSQI-evaluated sleep quality (*ρ*=-0.52, *p*=0.031) (Fig. 1A and B). Controlling for disease activity and age, PFC S_t_O_2_ had a significant negative correlation with RANTES levels (*r*=-0.56, *p*=0.004) (Fig. 1C). Mediation analysis identified RANTES as a significant mediator of the S_t_O_2_-PSQI relationship (*p*=0.020), accounting for 29% of the total effect.

**Conclusions:**

We found that CD patients have low PFC S_t_O_2_ associated with altered interoceptive awareness and poor sleep quality; changes potentially mediated, at least in part, through systemic RANTES (Regulated on Activation, Normal T-Cell Expressed and Secreted; a proinflammatory chemokine) signalling to the brain. Our novel findings suggest a potential link between low PFC oxygen, systemic inflammation, and adverse symptoms in CD. FD-NIRS may provide a non-invasive method to study the association between CD-associated systemic inflammatory changes and symptom burden.

A225 Table 1: Summary of StO2 Values

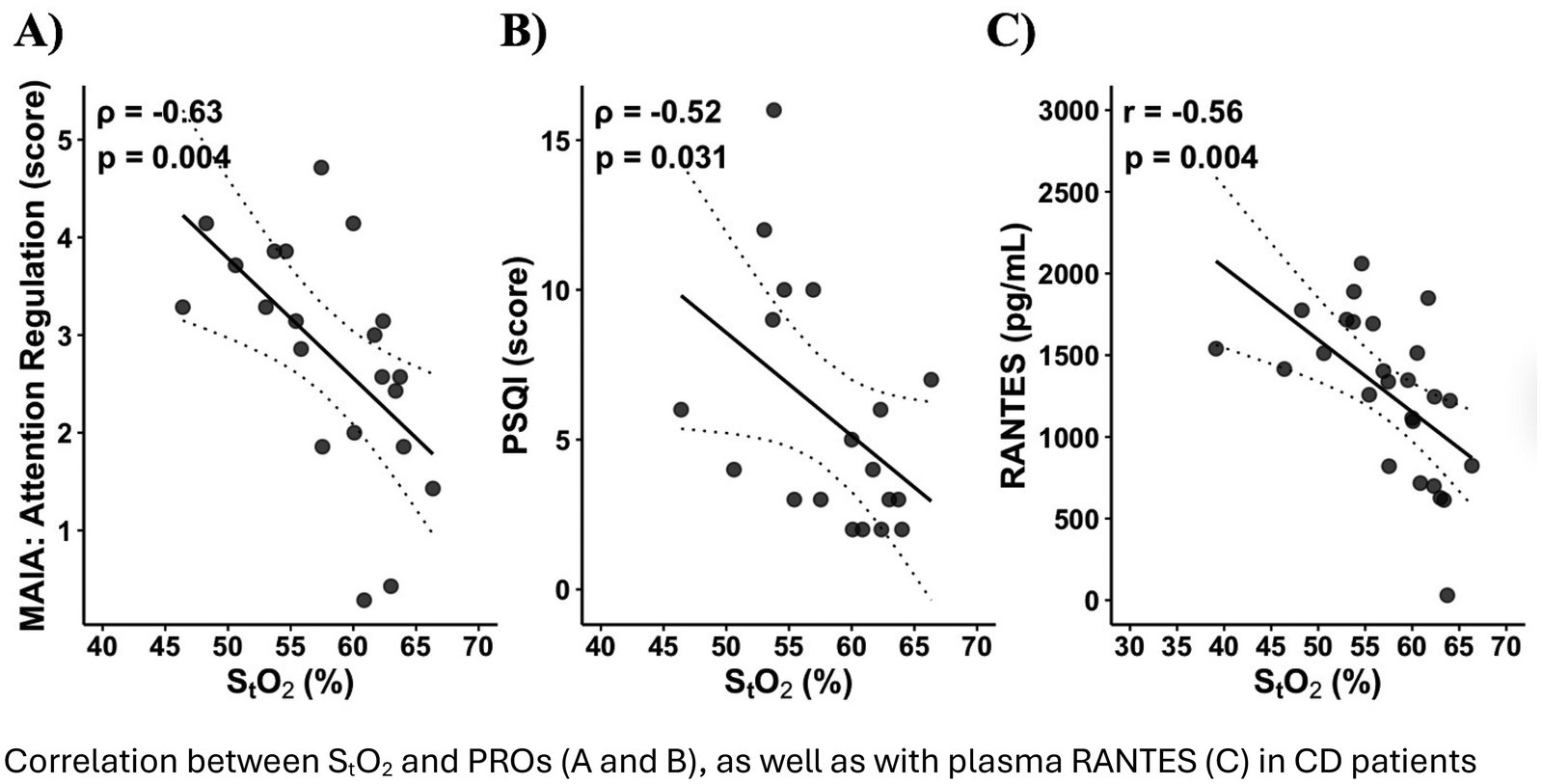

CIHR

**Table:** 

Group	*n*	*M±SD*
HC	19	62.7±4.02
rCD	16	56.9±5.44
aCD	10	58.0±7.69

